# Extraordinary disease-free survival in a rare malignant extrarenal rhabdoid tumor: a case report and review of the literature

**DOI:** 10.1186/s13256-017-1554-2

**Published:** 2018-02-17

**Authors:** Francesco D’Amico, Alessandra Bertacco, Maurizio Cesari, Claudia Mescoli, Giorgio Caturegli, Gabriel Gondolesi, Umberto Cillo

**Affiliations:** 1 Department of Surgery, Oncology and Gastroenterology, Hepatobiliary Surgery and Liver Transplantation, Padova University, Padova, Italy; 2 Department of Surgery, Division of Transplantation and Immunology, Yale University, New Haven, CT USA; 3 Department of Medicine-DIMED, Clinica dell’Ipertensione Arteriosa, University of Padova, Padova, Italy; 4 Surgical Pathology and Cytopathology Unit, Department of Medicine (DIMED), University Hospital of Padova, Padova, Italy; 5 Department of Surgery, Favaloro Foundation, Buenos Aires University, Buenos Aires, Argentina; 6 Hepatobiliary Surgery and Liver Transplantation Unit, Department of Surgery, Oncology and Gastroenterology, University Hospital of Padova, Via Giustiniani 2, 35128 Padova, Italy

**Keywords:** Extrarenal rhabdoid tumor, Rare colon tumor, MERT, Extended surgery, Literature review, Case report

## Abstract

**Background:**

Malignant extrarenal rhabdoid tumor of the gastrointestinal tract is rarely reported in the literature. It is characterized by poor prognosis and aggressive metastatic features.

A literature review evidenced only 19 cases, with poor outcome.

**Case presentation:**

We report a case of a colonic “pure” malignant extrarenal rhabdoid tumor with metastatic nodes in a 65-year-old Caucasian man. He was treated surgically with no recurrence, no adjuvant chemotherapy, and with 4-year survival without disease at the time of the submission of this article.

**Conclusions:**

We present an extraordinary case of long-term survival due to the extended surgical treatment.

We believe that the absence of organ metastasis at presentation is a positive prognostic factor, although pathology confirmed node involvement (13/38 positive) on microscopy.

## Background

A malignant rhabdoid tumor (MRT) is a subtype of Wilms’ tumor; first described by Beckwith and Palmer in 1978 [1]. It is most common in children, usually with a renal localization. Adult forms are rare, and they are characterized by a poor prognosis. However, similar lesions arising in soft tissue and other sites have been reported and referred to as malignant extrarenal rhabdoid tumors (MERTs), which have been accepted as a clinical-pathological entity of their own. They share common histology and immunophenotype, and can be mixed with different types of neoplasms (carcinomas, melanomas, and sarcomas). The extrarenal variants are very aggressive, and have most often been located in the central nervous system (CNS), liver, soft tissue, and colon [2]. Presentation in the gastrointestinal (GI) tract is rare and the prognosis of the affected patient is poor due to the aggressive nature of this disease. MRT is characterized by early diffuse metastasis, with death generally occurring within 6 months from initial diagnosis [3].

## Case presentation

A 65-year-old Caucasian man was admitted to Padova University Hospital for diffuse abdominal pain associated with generalized weakness, decreased oral intake, and weight loss of 20 kg in 2 months.

A physical examination at admission showed pale conjunctiva and skin, bilateral auscultation revealed clear lungs, and heart auscultation did not elicit any murmurs, rubs, or gallop.

An abdominal examination induced abdominal pain with rebound tenderness; bowel sounds were present in all quadrants. A right inguinal mass suggestive of lymphadenopathy was found on examination. The rest of the physical examination was unremarkable.

Patient history revealed ischemic cardiomyopathy treated with endovascular coronary revascularization, diabetes, and depression. Diverticulosis of the colon and sigmoid polyposis were reported in a colonoscopy performed the previous year. No family history of malignancy was reported. During the recovery, our patient underwent esophagogastroduodenoscopy (EGD), which was negative, and a colonoscopy, which showed an ab-extrinsic compression of the cecum.

**Fig. 1:**
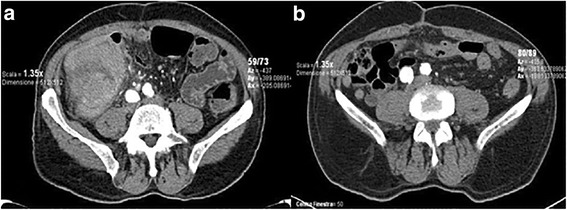
Computed tomography scan describing a mass involving cecum and ascending colon (**a**) and the absence of recurrence at a 4-year follow-up (**b**)

A total body computed tomography (CT) scan was performed, and it described a 10 × 8 × 10 cm mass invading the cecum and ascending colon (Fig. 1a) with lymphadenopathy. No metastasis was found. Tumor markers were negative.

Our patient underwent an elective right hemicolectomy with extended lymphadenectomy in the hepatogastric and aortocaval areas, and renal fat excision. The post-operative period was uneventful and he was discharged from the surgical unit on post-operative day 6.

**Fig. 2:**
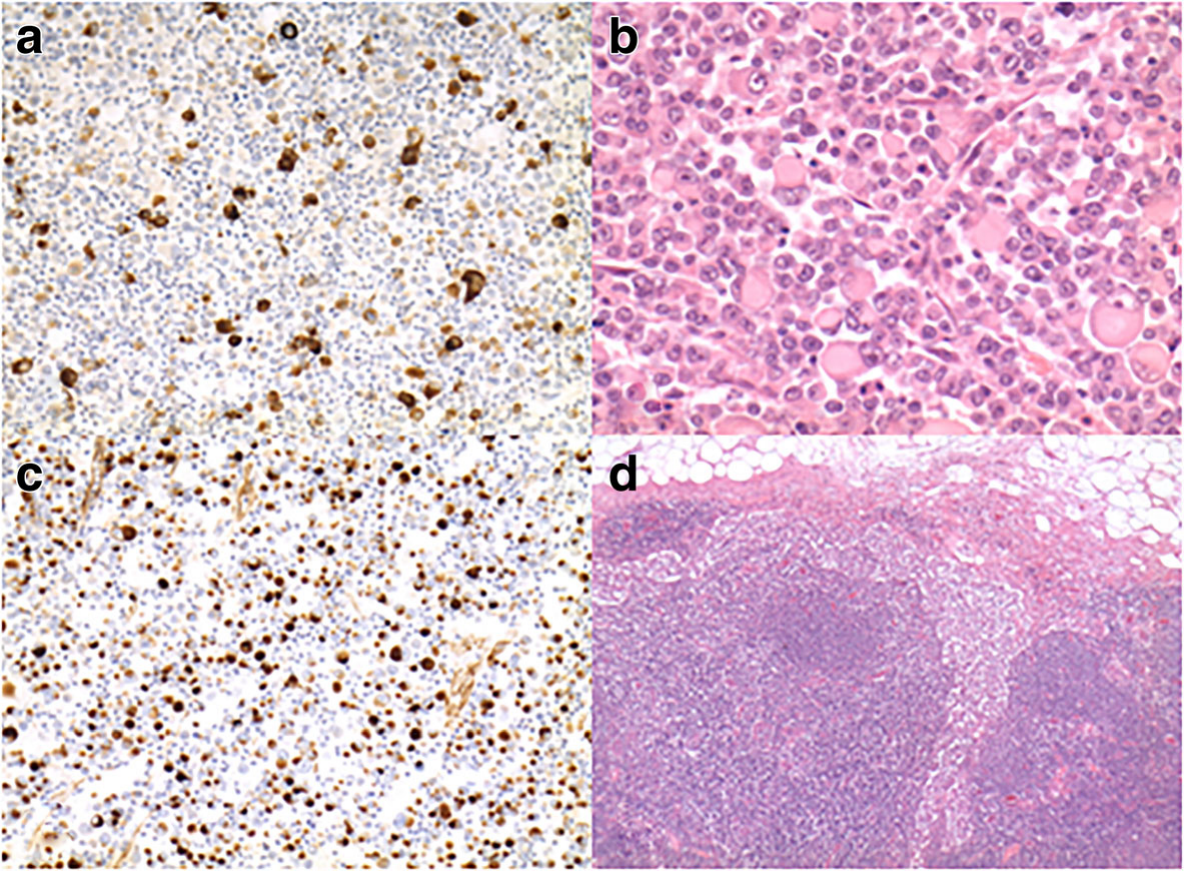
Immunohistochemistry gross pathology of our case. **a** MNF 117 negative (×20); **b** hematoxylin and eosin tumor cells (**c**) rhabdoid component, and positive for vimentin 50–70% (×20) (**d**) positive node (×5)

The pathologic diagnosis was rhabdoid tumor. The tumor stage was defined as T3N2M0 (13/38 positive nodes). Immunohistochemistry revealed cells positive for vimentin (Fig. 2) and epithelial membranous antigen (EMA), and negative for CK7, CD34, CD31, CD20, CD3, CD45, C-Kit, OCT-2. Immunostaining for integrase interactor 1 (INI-1) showed loss of nuclear expression, which is consistent with MERT. Fluorescence in situ hybridization (FISH) for the corresponding SMARCB1 was not performed in consideration of the immunohistochemical results. Our patient did not undergo adjuvant chemotherapy, and he is still alive and in good clinical conditions 48 months later, without recurrence (Fig. 1b).

## Literature review

We performed a systematic review of the literature regarding cases of colonic intestinal MERT. Case reports and center series were identified by PubMed “MeSH” search up to October 2016. Search items included: MERT, malignant rhabdoid tumor, colon rhabdoid tumor, rhabdoid colon cancer, and rhabdoid carcinomas. The cases obtained were from 19 manuscripts plus our case report, for a total of 20 patients to be analyzed.

A thorough search of the literature revealed 19 cases of primary rhabdoid tumor of the colon. Table 1 shows the clinical and histological characteristics of all cases of poorly differentiated carcinoma of the colon with rhabdoid features found in the literature. This entity seems to be a disease of older people with a mean age of 68 years at presentation; only four recent articles described the disease in young patients (<50 years). No gender predilection was evident (the male/female ratio was 10/9). The average size at presentation, which we calculated based on the tumor’s longest diameter, was 8 cm. The largest tumor was 15 cm and the smallest one was 4 cm. These tumors were distributed equally along the colon. Only four patients did not have identifiable organ metastasis or positive nodes at presentation. Fourteen patients had at least one positive lymph node invasion or distant metastasis. From the 19 cases in the literature, 11 cases had mixed histologic characteristics with adenocarcinoma and rhabdoid features, whereas the remaining eight cases had “pure” rhabdoid features (Table 2).

**Table 1:** Literature review of colonic rhabdoid tumors

Author	Age/gender	Site	Size (cm)	Treatment	Hystology	Outcome	Metastasis	CT
Chetty, *et al*. 1993 [15]	72 M	Cecum	6×5×2	R hemicolectomy	Composite	Dead (3 mo)	Liver, nodes	
Yang, *et al*. 1994 [16]	75 M	Trasverse	15×10×5	R hemicolectomy	Pure	Dead (2 wk)	Nodes	
Macak, *et al*. 1995 [17]	50 M	Rectum	6×5×3.5	Resection	Composite	*Not reported*	Nodes	
Marcus, *et al*. 1996 [18]	84 F	Trasverse	7	Resection	Composite	Alive (12 mo)	None	
Nakamura, *et al*. 1999 [19]	76 M	Cecum	14×8×6	R hemicolectomy	Pure	Dead (3 mo)	Liver, nodes	
Kono, *et al*. 2007 [20]	66 M	Cecum	13×13	R hemicolectomy	Composite	Dead (6 wk)	Nodes	
Lee, *et al.* 2013 [4]	62 M 83 F	Sigmoid colon Rectum	4×4×1 6.5×4.3	Low anterior resection Low anterior resection	Composite Composite	Alive (36 mo) Dead (1 mo)	Nodes Liver, lung, Nodes	Yes
Moussaly, *et al*. 2015 [21]	87 F	Trasverse	12×9.5×8.5	R hemicolectomy	Composite	Dead (2 mo)	None	
Mastoraki, *et al.* 2009 [22]	62 F	Descending colon	8×10×8	Resection	Pure	Dead (4 mo)	Liver	
Han, *et al*. 2010 [9]	23 F	Rectum	6×5	Miles resection	Pure	Alive (17 mo)	None	Yes + RT
Remo, *et al.* 2012 [5]	73 F	Right colon	10×4	R hemicolectomy	Composite	Dead (6 mo)	Nodes	Yes
Pancione, *et al*. 2011 [6]	71 F	Ascending colon	10×10	R hemicolectomy	Pure	Dead (8 mo)	Liver, peritoneum nodes	Yes
Agaimy, *et al.* 2014 [23]	79 M	Cecum	9×5×2	R hemicolectomy	Pure	Dead (6 mo)	1 regional node	
Romera Barba, *et al.* 2014 [24]	77 M	Descending colon	-	L hemicolectomy	Pure	Dead (2 mo)	None	
Baba, *et al.* 2014 [25]	45 F	-	-	Resection	Composite	Dead (6 wk)	Not reported	
Cho, *et al.* 2015 [26]	73 M	Cecum	4×x3	R hemicolectomy	Composite	Alive (1 mo)	Nodes	
Kalyan, *et al.* 2015 [7]	31 F	Cecum	7	R hemicolectomy	Composite	Dead (4 mo)	Nodes	Yes
Samalavicius, *et al*. 2013 [8]	49 M	Rectum	7	Total mesorectal excision	Pure	Dead (7 mo)	Nodes	Yes
D’Amico *et al*., present study	65 M	Ascending colon	10	R hemicolectomy	Pure	Alive (48 mo)	Nodes	

*CT* chemotherapy, *mo* months, *RT* radiotherapy, *wk* week

**Table 2:** Subgroup analysis (our case was excluded)

	Cases	Dead/max survival	Organ metastasis/ nodes in dead	CT, RT in dead	Alive/max survival	Organ metastasis/nodes in alive	CT, RT in alive	Survival literature review
Pure	8	7 / 7 mo	50% / 83%	28,5%	1 / 17 mo	0%, 0%	100%	12,5%
Composite	11*	7 / 6 mo	33% / 83%	28,5%	3 / 36 mo	0%, 67%	33,4%	30%

*CT* chemotherapy, *mo* months, *RT* radiotherapy

*One patient did not have survival reported in the literature review

All patients underwent surgical resection as the first line of therapy. The overall survival for this tumor, even after surgical intervention, seems to be limited, with a large majority of the patients surviving less than 6 months. Only three studies described a survival of 1 year; in these patients there was no distant metastatic organ disease at the time of presentation. Six patients received adjuvant chemotherapy, five of them were N1. One patient received 12 cycles of leucovorin, flurouracil, and oxaliplatin (FOLFOX) and he was still alive at the time the manuscript was written, surviving 36 months without recurrence or distant metastasis [4]. Another patient received a trial of capecitabine and oxaliplatin and had a 6-month overall survival [5]. A third patient received a trial of bevacizumab and cetuximab and had an 8-month survival [6]. A fourth patient received one cycle of 5-flurouracil, leucovorin, and oxaliplatin (mFOLFOX6), palliative radiosurgery to the rib, and three cycles of epirubicin, capecitabine and oxaliplatin (EOX), but she expired within 4 months of her diagnosis [7]. Samalavicius *et al.* treated their patient with FOLFOX4 and leucovorin, flurouracil, and irinotecan (FOLFIRI) regimen, with a survival of 7 months [8]. Han *et al.* described therapy with adjuvant radiotherapy (40 Gy) and chemotherapy (5-fluorouracil and oxaliplatin) [9].

## Discussion

Malignant rhabdoid tumors (MRTs) are aggressive neoplasms that were first described in young children with renal primary neoplasms [10, 11]. MRTs were later seen to arise at different locations, such as the central nervous system (CNS) [5], liver [6], genitourinary tract [7], and GI tract [8], known with the name of extrarenal rhabdoid tumors (MERTs). The histologic hallmark of MERTs is a centrically located and large nucleus, prominent nucleoli, abundant and eosinophilic cytoplasm, and paranuclear inclusions of intermediate filaments and abundant mitotic figures. Review of the pathologic characteristics of all cases available in the literature demonstrates that centric nuclei are the common feature among all MERTs.

These tumors are described as “composite” when the rhabdoid phenotype is mixed with another type of identifiable neoplasm and termed “pure” when the rhabdoid features are the only identifiable phenotype. When adenocarcinoma and rhabdoid tumor coexist, the rhabdoid features remained similar in all of them [4].

The histogenesis of MERT is unclear. Ota *et al*. [12] suggested that the rhabdoid tumor cells were derived from a primitive pluripotent cell that had the potential for a wide range of differentiation and accounted for the phenotypic heterogeneity observed in MERTs. In the literature review, we observed that the only patient alive in the “pure” subgroup had no involvement of lymphatic nodes or organ metastasis at the time of surgery, and underwent adjuvant chemotherapy and radiotherapy. In the “composite” subgroup, the patient with a longer survival had positive nodes and received chemotherapy (Table 2). This confirms the difficulty to define a prognosis based on histological findings and the absence of a standard therapeutic protocol, implying the relevance of performing an R0 resection in the first place.

The management of these tumors is usually surgical resection if the tumor is operable. The rare occurrence of MERT has made it complicated to establish adequate survival-improving protocols. Most of the reports indicate that MERTs are highly aggressive tumors, with more than 75% of patients dying within 6 months of the initial diagnosis [3, 4].

GI rhabdoid tumors are very rare. The majority of extrarenal rhabdoid tumors occur in children, while GI rhabdoid tumors are more common in the elderly population. Cytokeratin and vimentin are frequently found on immunochemistry.

Historically, all the rhabdoid colorectal cancers reported in the literature primarily affect older people, with ages ranging from 62 to 84 years of age and with no gender preference. Nevertheless, we found a few cases in young patients in our review.

Recent reviews suggested that these tumors are large and tend to be located in the transverse colon or proximal ascending colon [3, 4], however, our literature review highlighted a predominance in cecum involvement. Surgical tumor resection was adopted by all series as first-line treatment, but despite it, survival remained low with a median survival of 7 months. We noted that patients alive at the time of publication were patients without metastasis at presentation or with limited node involvement. Nevertheless, two of four patients with no macroscopic metastasis or positive nodes during the surgical intervention, expired in less than 2 months. Horazdovsky *et al*. concluded in their meta-analysis that surgery and actiomycin therapy may improve survival in MERT [13]. Beneficial results of adjuvant treatment have been reported for MERTs in the skin and CNS, but not in the liver. Chemotherapy did not seem to impact survival (Table 1); also, Pancione *et al.* [6] and Remo *et al*. [5] reported that adjuvant chemotherapy had no significant benefit. The role of adjuvant treatment for MERT in the gastrointestinal tract is unknown. Since extrarenal rhabdoid tumors are rare, no standard therapeutic pathway exists and no randomized trials that examine the role of chemotherapy combinations or addition of new drugs have been done. Also, the use of radiotherapy did not improve survival in adults with MRTs [14].

We report an extraordinary case of a patient with long-term survival due to the extended surgical treatment. We believe that the absence of organ metastasis at the presentation should be a positive prognostic factor, although pathology confirmed node involvement (13/38 positive) on microscopy. Since we performed an extended right hemicolectomy with lymphadenectomy (upto the main nodes), omentectomy, and removal of the perirenal fat, we believe that the combination of no organ metastasis and careful extended surgery could explain this extraordinary case of long survival. Our Department of Oncology did not propose chemotherapy for our patient due to the absence of a therapeutic protocol with proven efficacy in the literature for this rare tumor. The follow-up CT scans showed absence of disease.

## Conclusions

In conclusion, the current case report is, to our knowledge, the only case of a patient with colonic rhabdoid tumor with extended surgical resection with a survival of 4 years without recurrence or metastatic disease evidenced in control CT scans. All the published cases exhibit similar clinical-pathological features, as well as the characteristic histological features of sarcoma differentiation of rhabdoid cells, which appear to indicate aggressive biological behavior. Further investigations into this highly aggressive colonic carcinoma showing rhabdoid features are required in order to determine specific and effective treatment for this type of tumor.

## Abbreviations

CK: Cytokeratin
CNS: Central nervous system
CT: Computed tomography
EGD: Esophagogastroduodenoscopy
EMA: Epithelial membranous antigen
FISH: Fluorescence in situ hybridization
GI: Gastrointestinal
INI-1: Immunostaining for integrase interactor 1
MERT: Malignant extrarenal rhabdoid tumor
MRT: Malignant rhabdoid tumor
